# Development of an Intelligent Wind Erosion Monitoring System

**DOI:** 10.3390/s23239389

**Published:** 2023-11-24

**Authors:** Linhu Wang, Chengyu Li, Jianhui Lin, Siwen Ni

**Affiliations:** School of Technology, Beijing Forestry University, Beijing 100083, China; wanglinhu@bjfu.edu.cn (L.W.); li_chengyu1996@163.com (C.L.); 15652861990@163.com (S.N.)

**Keywords:** soil erosion, wind erosion monitoring system, high-precision calibration of load cell, six-axis acceleration sensor

## Abstract

Wind erosion monitoring is an important method for measuring soil erosion and desertification. However, the current wind erosion monitoring equipment has the disadvantages of low automation and low measurement accuracy. In this work, an intelligent wind erosion monitoring system is developed, which can automatically collect and upload information on sand and the environment. The structure of the mechanical parts is designed and optimized to reduce the measurement error caused by the windblown sand impact on the sample collection to improve the stability of the system. A specific scheme for the precision calibration of the load cell is developed and implemented. The jitter parameters of the load cell were determined using the JY61 six-axis acceleration sensor, and then the optimal scheme to eliminate the jitter error was determined by comparing two neural network models in MATLAB 2021a software, and the precision calibration of the load cell was completed. As a result, the system has a reliable mechanical structure and hardware system and a perfect error compensation processing scheme. In a certain period, the system can be fully automatic with stable operation. The field operation test of this system can meet the design requirements and improve the measurement accuracy of windblown sand wells.

## 1. Introduction

Desertification is one of the most serious environmental problems facing the world today [1]. Wind erosion causes a serious loss of fine-grained matter and nutrients in the soil, causing a coarser soil texture and significantly reducing soil organic matter and nutrient content, thus leading to a decline in soil quality and land productivity [2,3]. With the pursuit of a desired living environment in recent years, it is urgent to control soil wind erosion [4].

Throughout the global research on soil wind erosion, after a large number of wind tunnel simulations and field observations, researchers have proposed different wind erosion estimation, prediction, random, coupling, and dynamic models for different actual conditions [2], including some empirical or semi-empirical models.

Many studies have been carried out on horizontal sand dust fluxes, such as in Europe, China, Canada, Australia, and the United States, especially in arid and semi-arid regions. On Earth, this occurs mainly in deserts, on beaches, and in other sparsely vegetated areas, such as dry lake beds [5,6,7,8,9].

Meanwhile, the natural and human factors affecting the process of soil wind erosion were classified, and different types of wind erosion influencing factors were summarized [10], which promoted the monitoring and control of soil wind erosion. However, it is very important to verify the reasonableness of these models and influence factors and to obtain the actual data of wind-eroded soil through the sand collecting instrument in the process of soil wind erosion monitoring equipment. A sand collecting instrument is the key equipment for studying soil wind erosion, which is also an experimental instrument for directly acquiring soil wind erosion data. When soil wind erosion is carried out, most of the corroded material will fall back to the ground after floating at a certain height and distance, while a small part of the corroded material will be suspended in the wind and reach a far place [11]. A large amount of data can be obtained by setting the sand collecting instrument in different places and different heights of the monitoring site and collecting sand in corresponding positions.

In the 1940s, Bagnold designed the first instrument for observing soil wind erosion in the world, which is a vertical long-mouth sand collector. Subsequently, Merva and Peterson, Shao, Greeley, Fryrear [12,13,14,15], etc. optimized this instrument and the sand collection equipment could better adapt to different weather and wind speeds in the field [16]. Later, different researchers designed a variety of monitoring types of sand collection equipment [17], whose main research was to improve the mechanical structure to improve the sampling efficiency of the sand collecting instrument [18].

With the development of electronic components, automatic sand collecting instruments have begun to appear. In 2015, Ioannis Kosmadakis and others developed an automatic sand collecting instrument, but it was unable to monitor environmental information, and the design was too simple to consider the effectiveness of the equipment [19]. In 2019, Antonio Giménez and others presented a new wind tunnel design that enables a very simple analysis of soil wind erosion. This wind tunnel is designed to be used in any terrain and has an automatic data collection and analysis system. However, its structure has certain problems, leading to large monitoring errors, and it cannot operate outdoors for a long time [20]. In 2019, Cui Yazhen designed an automatic continuous weighing sand collecting instrument and found in field tests that jitter would cause measurement errors in the system, but did not provide a specific implementation plan to improve the detection accuracy [21]. In 2021, Jung-Rack Kim and others developed an approach to monitor a sequence of aeolian erosion in a sand desert area by fusing a set of remote sensing data. The scheme will be beneficial for the evaluation of combating desertification activities and the early warning of dust storm generations. However, they did not consider the actual field conditions [22].

In recent years, researchers have focused on the improvement of the automation of wind erosion monitoring equipment, but there are few studies on the measurement accuracy of monitoring equipment. Sand measurement is the most important part of the soil wind erosion and desertification process. Due to the insufficient collection accuracy of the current wind erosion research equipment worldwide, this work analyzed the reasons for the large deviation and eliminated the deviation between the measurement results of the weighing sensor in the sand collection device and the actual value caused by the effects of strong wind, large temperature differences, equipment jitter, and other factors in harsh field environments [23].

Firstly, based on the designed mechanical structure model, the measurement error caused by the wind pressure is analyzed and eliminated [24]. Then, the sensor calibration experiment, temperature compensation experiment, and jitter experiment of simulated natural wind were carried out to calibrate the weighing sensor, so as to improve the sand collection accuracy. Finally, the field experiment is completed to verify the accuracy of the monitoring system.

## 2. Composition and Function of Intelligent Wind Erosion Monitoring System

**Figure 1:**
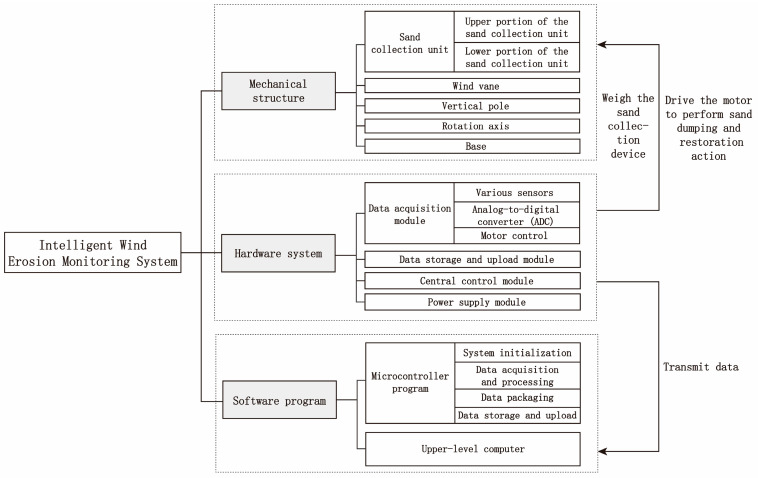
Intelligent wind erosion monitoring system overall composition structure diagram.

A wind erosion monitoring system consists of three parts: mechanical structure, hardware system, and software program, as depicted in Figure 1.

**Figure 2:**
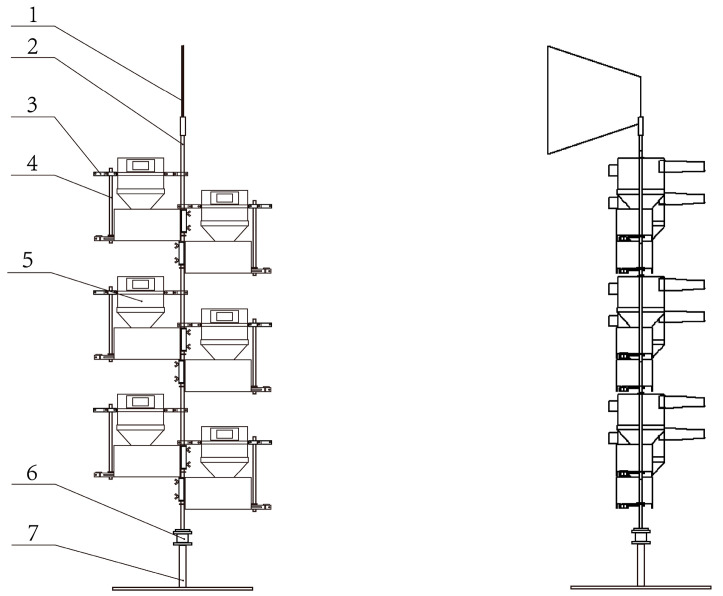
General mechanical structure drawing: (1) Wind vane; (2) Vertical rod; (3) Connecting rod; (4) Fixed rod; (5) Sand collecting unit; (6) Rotating shaft; (7) Base.

The mechanical structure of the wind erosion monitoring system is composed of one base, one vertical rod, one rotating shaft, one wind vane, one solar panel system with battery storage, four sand collecting units, and the corresponding number of supporting plates and fixtures. Each sand collecting unit is equipped with a hardware system and various collection modules. The overall mechanical structure of the equipment is shown in Figure 2. In application, the part below the rotating shaft of the system is buried underground, and the battery and its protection device are also buried underground as the base counterweight. A wind vane located at the top of the vertical rod drives the part of the monitoring system above the rotating shaft to rotate with the wind.

Long hours of automatic monitoring can be achieved by this system at the monitoring site. The hardware system controlled by STM32 could process the collected load cell data and the surrounding environment information data in two ways: local storage and upload Alibaba Cloud Internet of Things server display. In this system, the whole process of sand collection, weighing, sand collection device dumping after full collection, data storage, and uploading does not require manual operation in the monitoring site.

## 3. Experimental Section

Based on the considerations of utilizing a wind erosion monitoring system in challenging climatic conditions, this chapter’s research will be divided into two major parts. In the first part, a scheme to mitigate wind-induced errors in load cell measurements is formulated. High-speed winds can lead to substantial measurement inaccuracies and a reduced load cell lifespan. Therefore, a sand collecting unit was designed to reduce wind velocity within the structure. Mechanical and fluid simulations were conducted using SolidWorks 3D 2021 software and the ANSYS Fluent module. The positions of key components were optimized using the orthogonal experimental method to ensure the most effective drag reduction. Subsequently, ANSYS Fluent fluid simulations were performed to study pressure at the sand outlet under varying wind speeds at the air intake, thereby eliminating wind-induced errors. Finally, real-world verification was conducted in a laboratory setting to ensure the accuracy of the design and simulation, validating the system’s performance.

In the second part, a high-precision load cell calibration scheme was devised, encompassing four key sections. Initially, to address issues stemming from manufacturing variations and ensure consistent sensor outputs, load cell calibration was carried out by applying standard weights to establish the relationship between output and input. Next, to enhance measurement accuracy, a digital filter was designed for signal processing, targeting factors like system vibrations and noise through median filtering. Thirdly, as temperature significantly affects the load cell, a temperature compensation design was implemented. By simulating various temperature environments and employing cubic spline interpolation to determine the functional relationship between temperature and error, the high-precision calibration of the data was achieved. Lastly, considering the potential impact of external environmental factors on system stability and measurement accuracy, a jitter compensation design was implemented. A six-axis acceleration sensor was used to collect data, and neural network analysis was employed to eliminate errors caused by jitter. These experimental designs collectively aim to ensure that the load cell provides high-precision measurement data under various environmental conditions and circumstances. Specific experimental results will be further analyzed in Section 4 of this paper.

### 3.1. Design of Wind Pressure Error Elimination Scheme for Load Cell

The installation and applications of wind erosion monitoring equipment are mostly in desert areas with harsh climatic conditions and high wind speeds, and the monitoring system needs to constantly weigh the dust collected in the sand collection device through a high-precision single-point load cell. When the sand is blown into the sand collecting unit of the monitoring system, the large velocity impact may bring a measurement error to the high-precision single-point load cell, and greatly reduce the service life of the load cell. In this part, the mechanical structure of the upper part of the sand collecting unit is reformed, so as to reduce the speed of sand blowing into the equipment. Additionally, this analyzes and eliminates the weighing error caused by the pressure impact of the residual wind on the load cell.

The sand collecting unit in the mechanical structure was designed by SolidWorks 3D software. Finite element analysis on the key components and complete fluid simulation for the upper part of the collected speed reduction was conducted by the ANSYS Fluent module. Orthogonal experiments were carried out on the design positions of important components in the structure to determine the design scheme of the optimal collection of deceleration parts to ensure the best deceleration effect. The wind speed of the sand at the sand collecting intake was not 0, which had an impact on the load cell, and resulted in the weighing error. Through the ANSYS Fluent fluid simulation experiment, the pressure corresponding to the sand outlet at different air speeds of the intake was determined and the error function was calculated. The experimental measurement was carried out on the no-load sand collecting unit in the laboratory to verify the validity of the calculated error function.

#### 3.1.1. The Design of the Sand Collecting Unit and the Orthogonal Experimentation for the Positioning of Parts in the Collection and Deceleration Part

**Figure 3:**
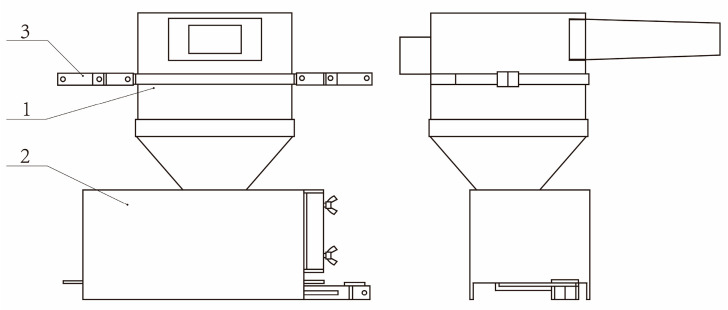
Sand collection unit: (1) Collection and deceleration part of the sand collecting unit; (2) Data collection part of sand collecting unit; (3) Fixed steel strip.

The smallest unit in the mechanical structure is the sand collecting unit, and there are four sand collecting units in the monitoring system. The function of the sand collecting unit is to collect sand automatically, weigh sand, and other related environmental parameters. The sand collecting unit adopts the upper and lower separation structure and adopts all-steel casting to improve reliability, which consists of the collection and deceleration part of the sand collection and deceleration part and the data acquisition part, as shown in Figure 3. The collection and deceleration of sand are completed by the collection and deceleration part. The automatic weighing of the collected sand, monitoring the environmental parameters, and storing, uploading, and dumping with full load are completed by the data collection part.

**Figure 4:**
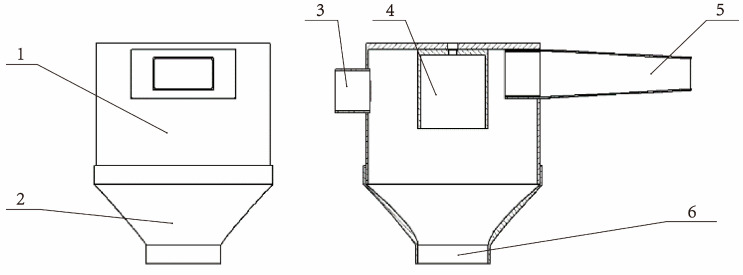
The collection and deceleration part of the sand collection unit: (1) Main sand pipe; (2) Collecting funnel; (3) Tail outlet; (4) Cylindrical diverter; (5) Air intake; (6) Sand outlet.

The collection and deceleration part of the sand collection unit is composed of an air intake, a main sand pipe, a cylindrical diverter, a tail outlet, and a collection funnel, as shown in Figure 4. When the wind erosion monitoring system works, the wind speed decreases gradually because the cross-sectional area of the air intake increases continuously. The cylindrical diverter can make the wind sand naturally divide into two strands, flowing from both sides of the cylindrical diverter, forming a convection behind the cylindrical diverter, making the wind speed lower again. After that, under the influence of the main sand pipe and the cylindrical diverter, most of the wind is discharged through the outlet, and the decelerated wind sand falls into the collection funnel through the sand outlet and flows into the data collection part at the lower part of the sand collection unit for weighing.

**Figure 5:**
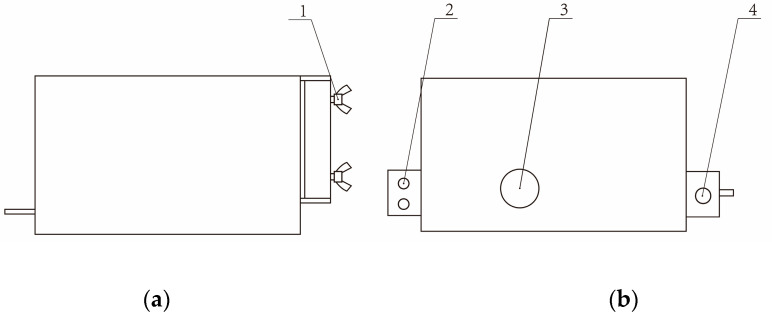
Stainless-steel case: (**a**) Front view; 1. Tightening screw; (**b**) Top view; 2. Fixing screw hole; 3. Round hole; 4. Fixing screw hole.

**Figure 6:**
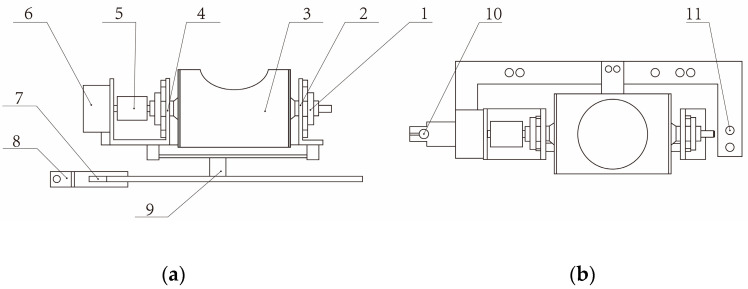
Sand collecting and processing equipment: (**a**) Front view; 1. Bearing; 2. Sand collecting device bracket; 3. Sand collecting box; 4. Stepper motor bracket; 5. Coupler; 6. Stepper motor; 7. Support plate; 8. Mechanical fastener; 9. Load cell; (**b**) Top view; 10. Fixing screw hole; 11. Fixing screw hole.

The data collection part of the sand collecting unit is mainly composed of the control circuit board, the sand collecting and processing equipment, and the environmental monitoring unit. All the components are wrapped in a stainless-steel case. The side of the stainless-steel case is equipped with a fixing device, and tightening screws allow the sand collecting unit to be securely fixed at any position along the vertical rod, facilitating researchers in collecting wind erosion data at different heights, as shown in Figure 5. Above the case, there are circular holes through which wind-blown sand, after being collected and decelerated in the upper part of the sand collecting unit, flows through the sand outlet and stainless-steel holes into the sand collection and processing equipment in the data collection part. The components of the equipment are shown in Figure 6. The stainless-steel case and the sand collection and processing device are secured together through two fixed screw holes which are connected by screw bolts.

When the sand collecting unit is working, the initial weight is peeled first, and the load cell weighs the sand collecting device at regular intervals. The microcontroller collects the weight data in real-time and controls the stepper motor to rotate the sand and complete the reset procedure if the sand collecting box is at risk of overflowing.

**Figure 7:**
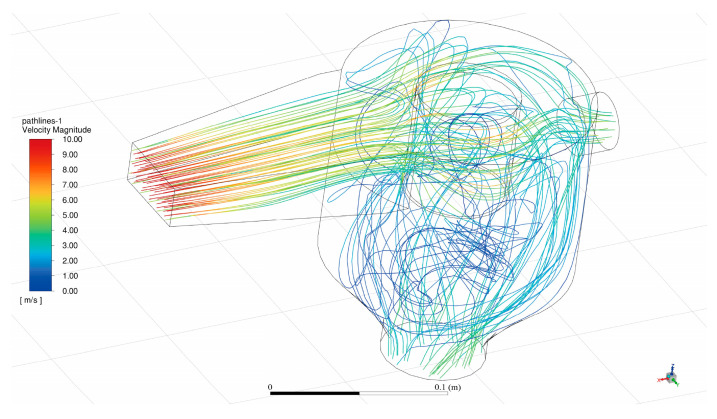
Streamline diagram.

To verify the application potential of the sand collecting unit, the internal fluid domain of the collection deceleration part was drawn via SolidWorks software, and the drawn model was imported into ANSYS Fluent Mesh for grid division. The mesh was constructed by the tetrahedral method, the growth rate was 1.2, the average orthogonal mass of the mesh was 0.75164, and the mesh quality was good. The divided mesh is imported into ANSYS Fluent for the next setting. The fluid domain is selected as air, and it is regarded as incompressible fluid at a low flow rate with the K-ω SST model as internal flow. The intake is selected as the speed inlet, the two exits are selected as the pressure outlet, and all the walls have no slip. Coupled was chosen as the calculation method, and the momentum, turbulent kinetic energy, and specific dissipation rate were all selected as the second-order upwind scheme. The calculation was started after the initialization was completed, and the results were visualized. The simulated flow diagram is shown in Figure 7 (when the intake speed is 10 m/s).

In Figure 7, different wind speeds are represented by corresponding colors. The highest wind speed is indicated by the color red at the air intake. At this point, the windblown sand enters the sand collecting unit system through a wedge-shaped air intake, designed to effectively guide and collect windblown sand. The wedge-shaped structure aids in the initial formation of streamline flow and partial reduction of wind speed, which is more conducive to the collection of sand particles and reduces erosion and wear on the internal structure of the sand collecting unit.

Subsequently, the sand-carrying airflow reaches the main sand tube, where it is divided into two parts by a cylindrical diverter. The cylindrical diverter, a cylindrical physical obstacle, splits the airflow into two directions, further reducing the wind speed and thereby enhancing the efficiency of sand collection. At this point, the color of the airflow in the streamline diagram has shifted predominantly to yellow–green. Behind the cylindrical diverter, there is a convection phenomenon in the airflow. In reality, local fluid circulation may occur due to uneven wind speed and temperature distribution. Convection helps further mix and decelerate the airflow.

After all these processes, the wind speed is significantly reduced, as evident in the streamline diagram where the airflow lines are predominantly blue–green. The sand particles, carried by the low-speed airflow, ultimately drop through the sand outlet to the lower half of the sand collecting unit, where the data collection section is located, for weighing processing. This step is the ultimate goal of the entire system design, aiming to collect as much sand as possible.

The design of the entire process takes into account the dynamic characteristics of air blown sand and how to efficiently collect sand while maintaining high energy efficiency. Through carefully designed physical structures, the system can control wind speed and effectively separate sand particles from the sand-carrying airflow to achieve the desired collection effect.

There exists windblown sand deceleration through the correlation analysis of the sand collection and the deceleration of the sand collecting unit in the above section. However, due to the complexity of its constituent components, this section will mainly conduct orthogonal experiments on the design positions of important components in the design to ensure the best deceleration effect.

Taking the wind speed of the sand outlet as the criterion, orthogonal optimization experiments were carried out based on five factors: the size of the air intake, the diameter of the cylindrical diverter, the distance between the top point of the tail outlet and the highest point of the equipment, the diameter of the tail outlet, and the diameter of the sand outlet. The parameters were set equidistant within the scope allowed by the whole structure. The five-factor, four-level orthogonal experimental design was carried out, as shown in Table 1, to optimize the design scheme of the sand collection and the deceleration part of the sand collecting unit.

#### 3.1.2. Wind Pressure Simulation Experiment Based on ANSYS Fluent

**Figure 8:**
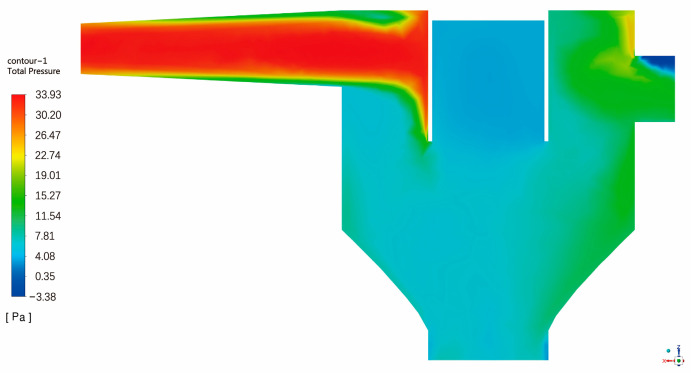
Total pressure distribution.

Through the ANSYS Fluent fluid simulation experiment, the surface integral of velocity and the pressure of the two outlets of the collected deceleration part were carried out, respectively, to obtain the average velocity of the outlet and the relative total pressure under different wind speeds of the intake. The simulated internal pressure diagram is shown in Figure 8 (the intake wind speed is 10 m/s). The experiment can simulate the relationship between the wind speed of the air intake, the tail outlet, and the sand outlet. At the same time, the pressure at the sand outlet can be obtained for different wind speeds at the air intake. After obtaining the real-time wind speed at the air intake through the anemometer, the pressure value of the wind force acting at the sand outlet for different wind speeds can be obtained by comparing the simulation results. Therefore, the error of wind pressure in this part can be eliminated.

#### 3.1.3. Wind Pressure Error Elimination Verification Experiment

To verify the accuracy of the pressure value obtained by ANSYS and the Fluent fluid simulation experiment, in a constant temperature laboratory, a large industrial fan was used to generate wind, and a monitoring system was set up. The monitoring system was equipped with a three-cup wind speed sensor that can measure the wind speed at the intake in real time. Simultaneously, to prevent system jitter, caused by a strong wind, from causing deviations affecting the accuracy of the experiment, only the sand collecting unit of the monitoring system was used for the experiment, and the sand collecting unit was kept fixed throughout the experiment. After the experiment began, the reading data of the wind speed sensor and the load cell under different wind forces were collected. The piecewise function corresponding to the pressure values of different load cells at different intakes in the simulation experiment was calculated and written into the collection program of the sand collecting monitoring system. With the real-time wind speed, different pressure compensation values can be calculated and the measured values of the load cells can be added and subtracted before output. From 20:00 on 20 May 2023 to 21:00 on 20 May 2023, the value of the wind speed sensor of the system and the compensated modular output value of the load cell of the sand were collected every 1 s. The wind speed of the industrial fan can be adjusted on eight levels, and the test wind speed close to the fan blade at the highest level can reach 18 m/s; simultaneously, the distance between the sand collecting unit and the fan blade can also be adjusted. Based on the above experimental conditions, a wind speed of 1 m/s in 0–18 m/s can be obtained. After compensating the output of the load cell for different wind speed values, the final output value of the analog-to-digital converter can be obtained.

### 3.2. Design of High Precision Calibration Scheme for Load Cell

**Figure 9:**
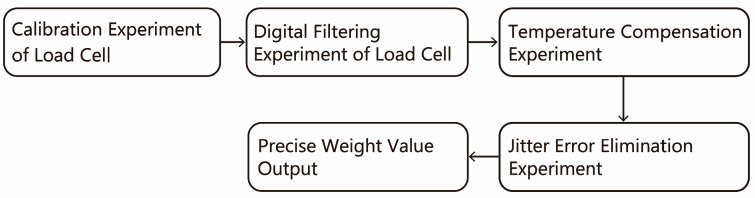
Flow chart of implementation of high precision calibration of load cell.

The high-precision calibration scheme of the load cell consists of four parts, and the implementation process is shown in Figure 9.

#### 3.2.1. Calibration Experiment of Load Cell

The principle of the load cell is converting the pressure into an electrical signal output, while errors exist in the output signals because of the construction difference of load cells. Therefore, it is needed to calibrate the sensor to determine the corresponding relationship between the output and input of the sensor. The pressure of the standard weight is applied to the load cell according to a certain step length, and the average value is taken as the calibration standard value to calibrate the load cell in the four sand collecting units.

#### 3.2.2. Digital Filtering Design of Load Cell

In the process of system operation, due to the system vibration and circuit interference, and the weak noise generated by the load cell, it is necessary to use a digital filter to reprocess the signal to improve the measurement accuracy [25,26,27].

The designed system is placed in the laboratory in a windless, unloaded, and normal temperature state to collect the AD output values of the load cell and obtain the original output values of the analog-to-digital converter. The output values are saved for comparison to evaluate the effectiveness of the digital filtering experiment.

In the single-chip computer program, the results of adjacent AD conversion for N times were collected by the software median filter [28], and then the arithmetic average value of the N data was calculated. It can be concluded as follows, the effect of using the median filter is proportional to the arithmetic average queue length, and the more data in the queue, the more stable the output weighing result will be. However, if the queue length of the median filter is increased, the reaction speed of the whole system will decrease, the CPU will consume too many resources, and the execution speed of the program will also decrease significantly. The conversion speed of the HX711 analog-to-digital converter is 80 sps (sample per second). When each bit is added to the filtering team, the system dynamic delay is 12.5 ms accordingly. Considering that the sand collection data of the whole continuous process needs to be saved, if too much data is calculated, the speed of the system refresh will decrease, which is unacceptable in the occasion of obtaining accurate and continuous data, such as the acquisition of wind sand. After the above analysis, the arithmetic average of the queue length of 5 is adopted, and the last 4 digits of the AD output value after the median filtering are discarded. This allows us to obtain relatively smooth data.

#### 3.2.3. Temperature Compensation Design

Since the resistivity, piezoresistive coefficient, elastic modulus, and other factors of the load cell material will change with temperature, errors may increase when the temperature changes. To obtain high-precision weight data, it is necessary to find the functional relationship between the sensor output and the ambient temperature, to minimize the error caused by the temperature [29].

Considering the laboratory conditions and the ambient temperature in practical application, the simulated ambient temperature test range in this temperature compensation experiment is −20 °C to 60 °C. In the experiment, the data acquisition part of the monitoring system was placed in the freezer to cool down or the dryer to heat up, and the pressure was selected as standard weights of 0 g (no additional load), 5 g, 10 g, 20 g, 50 g, 100 g, and 200 g for the experiments. The pressure from −20 °C to 30 °C and 30 °C to 60 °C were measured in a freezer and an oven, respectively. After measuring one set of pressure data, the load cell was set aside for 24 h until it was completely restored to room temperature for the next measurements. During the experiment, the ambient temperature of the system and the output value of the analog-to-digital converter of the sand collecting unit were collected every second. The temperature collection was accurate to one decimal place, and the precision range of the load cell was 6 decimal places.

The experimental results show that the relationship between pressure values and temperature is not simply linear; therefore, the curve reflecting the relationship between the input and output of the load cell should be selected to compensate for the error of the load cell. It has been decided to employ linear interpolation. In this method, the function segments are composed of several small intervals, thus requiring an adequate number of samples to enhance computational accuracy. The most widely used interpolation method is multinomial spline, and the cubic spline interpolation function in multinomial spline is the most widely used method. It is connected by small ranges of cubic polynomials, the real measured value is generally set at the connection and the second derivative is continuous, so the calculated function is a smooth continuous whole.

The selection of interpolation points in the cubic spline interpolation function is important, and the measurement range was divided into the same step length. The density of interpolation points directly affects the compensation effect and workload. Generally, the number of interpolation nodes were selected at about 10~25. In the temperature range of −20 °C to 60 °C, an interpolation was set every 5 °C and the arithmetic average of the weight measured by the load cell at the interpolation temperature was taken as the standard value of the current interpolation point. The final cubic spline interpolation function consisted of 16 sections of cubic polynomial smooth contact. The cubic interpolation spline function was calculated by MATLAB, with the coefficients in each polynomial, and the specific function expression of each curve can be obtained. After obtaining the ambient temperature, it can be substituted into the piecewise function expression to calculate the measurement error of the load cell at this temperature. By performing mathematical operations with this error and the measured value of the load cell, the true weight value at that time can be determined. Since the individual characteristics of each sensor are different, it is necessary to conduct experiments separately and calculate their respective function expressions.

#### 3.2.4. Jitter Compensation Design

During system operation, the system may jitter violently due to strong winds in the field, and there is an angle between the transmitted force and the stress axis of the sensor due to the tilt of the system, resulting in unreal force, etc. The jitter compensation of the load cell is adopted to eliminate the deviation between the measured value of the load cell and the real value [30].

The JY61 module was applied in the system jitter information measurement, which is the combination of the MPU-6050 six-axis acceleration sensor and STM8 chip; the user can directly read the acceleration, angular speed, and angle information during the movement through the serial port. Since the chip is integrated with the voltage regulator circuit and Kalman filter, the chip measurement accuracy is high. The attitude space of the sand collecting unit was defined, and a Cartesian coordinate system model was established. The vertical upward direction was defined as the positive direction of the Z axis, the direction away from the rotating rod was defined as the positive direction of the axis, and the front of the device was defined as the positive direction of the Y axis. In addition, the Euler angle in this coordinate system, pitch angle, roll angle, and yaw angle, are defined accordingly.

In this experiment, the monitoring system was set up in a constant temperature laboratory, and a large industrial fan was adapted to simulate the natural wind, and the jitter error of the load cell under the action of unapplied load and strong wind under different loads was collected. From 20:00 on 25 May 2023 to 23:00 on 25 May 2023, the collected values of the built-in six-axis acceleration sensor and the output values of the analog-to-digital converter of the sand collecting unit were collected every second. Due to the irregular jitter of the system, the experiment was conducted to simulate the maximum wind speed in the field. Six groups were collected under different weights in 180 min. A total of 10,893 sets of data were obtained, and X-axis angle, Y-axis angle, X-axis acceleration, Y-axis acceleration, X-axis angular velocity, Y-axis angular velocity, and the deviation between the output of the load cell and the standard weight at the corresponding time were collected.

The obtained data was analyzed in MATLAB using two types of neural networks. Among them, BP (Back Propagation) neural network is a multi-layer feedforward network trained according to the error reverse propagation algorithm, which was used to solve the minimum error. The BP neural network model includes an input layer, a hidden layer, and an output layer. In the process of network training, by comparing the difference between the output value and the expected value, the backpropagation algorithm is used to train and adjust the weights of several neurons in the hidden layer repeatedly, so that the final output value is close to the expected value and the error is reduced. Genetic Algorithm-Back Propagation (GA-BP) neural network model combines the advantages of Genetic Algorithm and neural network model and optimizes the initial weight and threshold of the neural network. The individual fitness value is calculated via the fitness function, and the individual corresponding to the optimal fitness is found via selection, crossover, and mutation operations, making its modeling more stable. The neural networks in this work are all three layers.

The parameters of the six-axis acceleration sensor are used as input and the reading values of the load cell as output. The neural network is used to calibrate the output of the load cell under jitter conditions.

#### 3.2.5. Experiment and Verification of Calibration Compensation

This section measures the overall accuracy of the optimized monitoring platform. In this experiment, the monitoring system was set up in the thirty Li wind area of Xiaocaohu in Turpan City, Xinjiang Uygur Autonomous Region, one of the three famous tuyeres in Xinjiang Standard weight pressure was carried out with M1 grade steel chromium-plated weights. The monitoring temperature ranged from 27 °C to 41 °C from 17:00 on 2 June 2023 to 23:00 on 2 June 2023. The measured wind speed was 0–17 m/s, which meets the environmental requirements of field application and conforms to the experimental measurement standards. At the beginning of the experiment, the ambient temperature, wind speed, six-axis acceleration sensor, and the output value of the collection unit analog-to-digital converter were collected every 2 s. Four sand collecting units were used to collect four groups of data under different pressures in the field environment; each group took 360 min. A total of 84,000 valid data sets were obtained. By analyzing the obtained data, we can determine whether the overall calibration design scheme for the load cell is reasonable.

## 4. Result

Section 2 provides a comprehensive discussion of the optimizations and enhancements necessary to improve the monitoring accuracy of a wind erosion monitoring system in harsh climatic conditions. This section will analyze and present the elimination scheme for the load cell wind pressure error and the experimental results of various calibration schemes. Each step corresponds to the theoretical design presented in Section 2 and is validated for its practical performance.

This section encompasses a series of experimental results aimed at enhancing the accuracy and reliability of the load cell under specific environmental conditions. Initially, through orthogonal experiment analysis of the drag reduction component of the sand collecting unit, the optimal structural parameters for reducing wind speed at the sand outlet were determined. Subsequently, ANSYS Fluent simulations were employed to further elucidate the pressure effects on the load cell due to wind speed. The model’s prediction of pressure values was verified to be accurate, affirming the effectiveness of our compensation function in mitigating wind pressure errors. When assessing the load cell’s linearity, all selected sensors exhibited excellent linearity and nearly perfect fits. To address sensor reading noise, we implemented a digital filter, resulting in a more stable AD conversion output.

Temperature compensation for the sensors utilized cubic spline interpolation, resulting in efficient compensation with weight output fluctuations within ±0.2 g. A comparison between the neural networks (BP and GA-BP) indicated that the latter provided higher prediction accuracy in the analysis results, significantly reducing errors in the sensor readings. Finally, a comprehensive system test was conducted in the 30 li wind zone of Xiaocaohu in Turpan City. Despite temperature fluctuations and strong winds causing system vibrations, the experimental results demonstrated that the output of our intelligent wind erosion monitoring system remained stable and accurate. Through our calibration and compensation techniques, the system’s overall measurement stability improved by 32%, and the measurement accuracy improved by 38%, confirming the effectiveness of the high-precision calibration design scheme for the intelligent wind erosion monitoring system.

### 4.1. Analysis of the Experimental Results for Wind Pressure Error Elimination

#### 4.1.1. Analysis of the Results of the Orthogonal Experiment for the Positioning of Parts in the Collection and Deceleration Part

The orthogonal experiment results of the simulation design of the sand collection and the deceleration part of the sand collecting unit are shown in Table 2 and Table 3, where the variables (A/B/C/D/E) correspond to the factors associated with the positions of the five components in Table 1. The numbers in Table 2 represent four different levels of design in Table 1. This table presents results based on the detection indicator of the average wind speed at the sand outlet. In Table 3, K1–K4 and k1–k4 are the sum and mean of the measurement results in levels 1–4. R is the range, which is the difference between the maximum and minimum in the k value [31]. According to the R-value, the factors affecting the intake wind speed are E > D >A > B > C, that is, the diameter of the sand outlet has the greatest influence on the wind speed, followed by the diameter of the tail outlet, and then the section size of the intake, and the diameter of the cylindrical diverter, while the distance between the tail outlet and the top of the device has the least influence. Further, the k1–k4 values are determined as follows: to obtain a lower outlet wind speed, the optimal design scheme for the collection speed reduction part of the sand collecting unit is A_3_B_2_C_2_D_4_E_4_, the section size of the intake is 50 mm × 25 mm, the diameter of the cylindrical diverter is 60 mm, the distance between the tail outlet and the top of the device is 39 mm, the diameter of the tail outlet is 33 mm, and the diameter of the sand outlet is 60 mm. According to the above optimal scheme, the sand collection and deceleration unit of the sand collection part is designed, and the simulation results show that the sand outlet wind speed is 3.08 m/s. Compared with the A_1_B_4_C_4_D_4_E_4_ sand outlet wind speed of 3.45 m/s in the orthogonal table, A_3_B_2_C_2_D_4_E_4_ is the optimal design scheme.

#### 4.1.2. Analysis of the Magnitude of the Pressure Values Acting on Load Cell at the Sand Outlet

After the ANSYS Fluent fluid simulation experiment, the results are summarized and concluded. The average wind speed at the sand outlet, the average wind speed at the tail outlet, and the relative pressure at the sand outlet are obtained at different wind speeds at the air intake. Please refer to Table 4 for detailed parameters.

**Figure 10:**
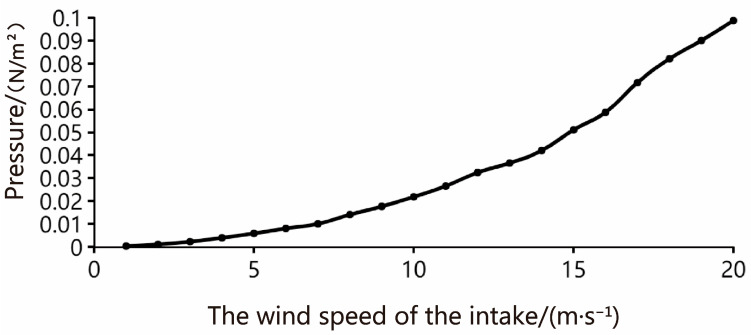
The relation curve between the air speed of the intake and the pressure of the load cell.

With the real-time wind speed value of the intake, the wind speed and pressure of the sand outlet can be obtained by referring to the table. From the previous analysis, the cross-sectional area of the sand outlet is set as 0.0028 square meters, and the pressure value of the load cell can be obtained by calculating the formula: pressure = pressure × force area (F = P × S, in Newton). The predicted value of the pressure on the load cell under different wind speeds of the intake is shown in the broken line in Figure 10.

#### 4.1.3. Display of Wind Pressure Error Elimination Effect

**Figure 11:**
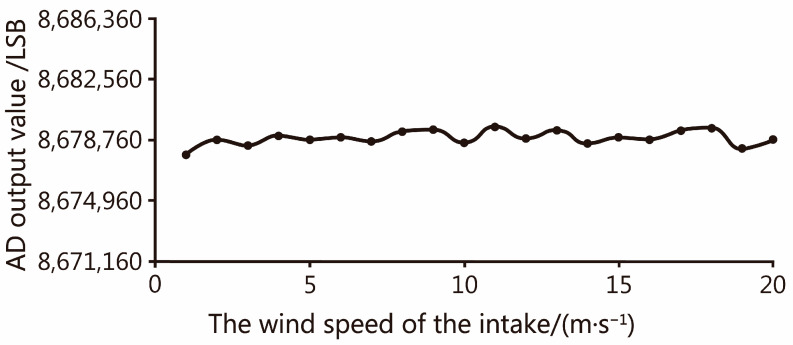
The relation curve between the air speed of the intake and the output of A/D converter.

According to the ANSYS Fluent fluid simulation experiment, the pressure values experienced by the load cell at different air intake wind speeds can be obtained. The final output of the analog-to-digital converter is recorded after different compensation of the output of the load cell under different wind speeds, and the measured value under the same wind speed is averaged as the final result, as shown in Figure 11. The final output results show that the prediction effect of the model used in this section is good, the predicted pressure value is accurate, the sand outlet air pressure can be eliminated by compensation function, and the experimental results are in line with expectations.

### 4.2. Analysis of the Experimental Results for High-Precision Calibration of Load Cell

#### 4.2.1. Linearity Characteristics of Load Cell

**Figure 12:**
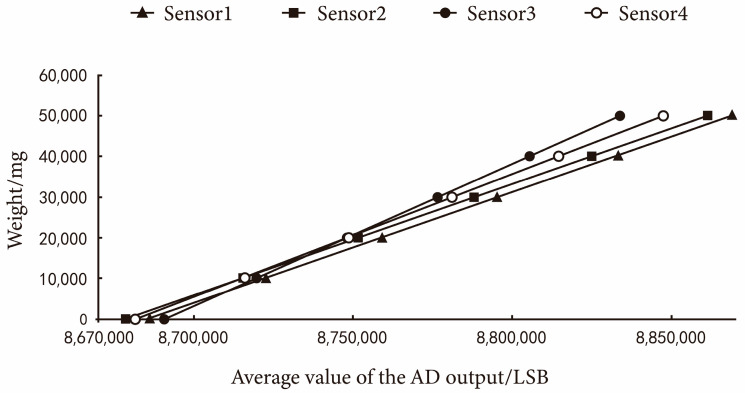
AD and counterbalance mass average scattergram.

Taking the average AD output value as the horizontal coordinate and the standard weight of pressure as the vertical coordinate, the curve is drawn as shown in Figure 12. The model chosen in this design is the Adachi-NA6 series 2000 g high-precision single-point load cell with good linearity and the fitting curve is 1.

#### 4.2.2. Analysis of the Digital Filtering Results for the Load Cell

**Figure 13:**
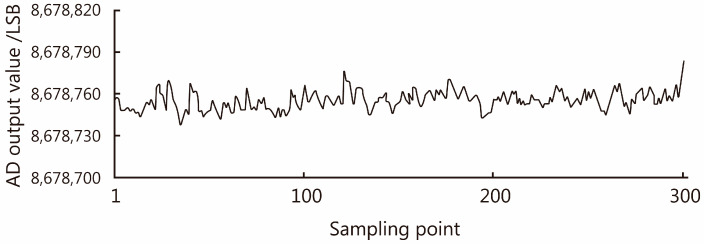
AD data of sand collection unit.

**Figure 14:**
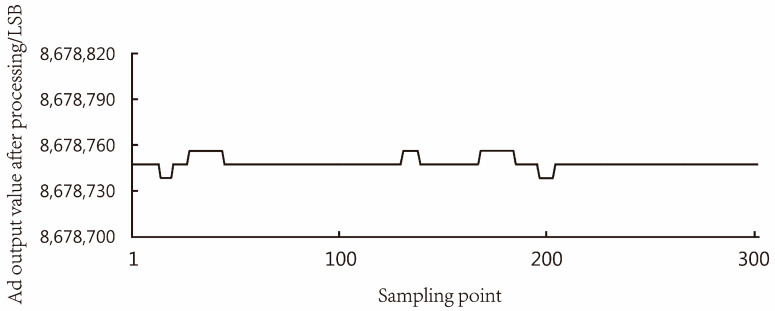
Data after median filter.

Before the digital filter design for the load cell, a set of data of the original output values of the analog-to-digital converter was collected, as shown in Figure 13, with significant fluctuations in the original output values. The output of the load cell after digital filtering is shown in Figure 14, which can meet the AD conversion output values tending to be stable under no-load conditions, helping to improve the accuracy of subsequent measurements.

#### 4.2.3. Using Cubic Spline Interpolation Function for Temperature Compensation of the Load Cell

**Figure 15:**
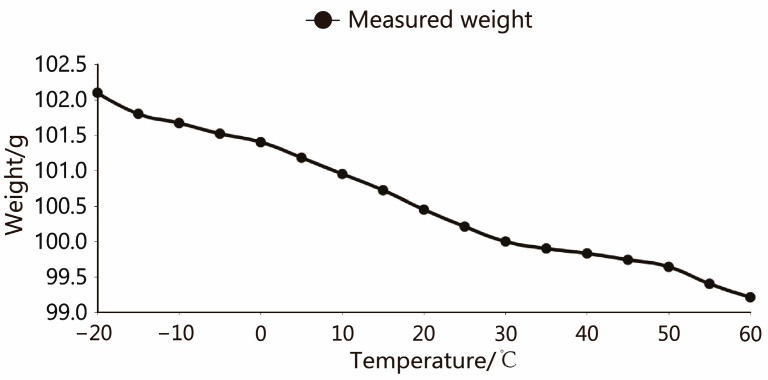
Temperature and weight output curve.

The pressure values measured at the same temperature are displayed using the arithmetic average value. The fluctuation curve of the load cell along with the temperature is shown in Figure 15 at the standard weight of 100 g. All AD output values were converted to weight grams for ease of presentation.

**Figure 16:**
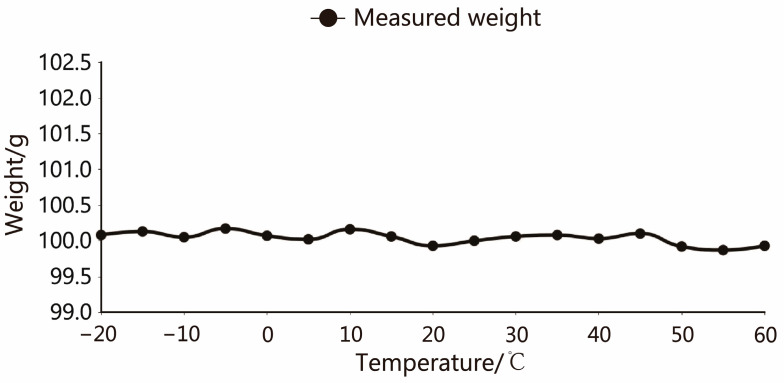
After compensation, the temperature and weight output curve.

The cubic interpolation spline function is calculated by MATLAB, with the coefficients in each polynomial, and the specific function expression of each curve can be obtained. Since the individual characteristics of each sensor are different, it is necessary to conduct experiments separately and calculate their respective function expressions. Figure 16 shows the final output result after Cubic spline interpolation functions for the No. 1 load cell. The weight output fluctuation range is ±0.2 g, and the compensation effect is reasonable.

In the following experiments, we found that when different loads were applied to the load cell, the influence of temperature on the load cell was consistent, and the effect of using the same cubic spline interpolation function was consistent.

#### 4.2.4. Comparison and Application of Two Neural Networks in Eliminating Jitter Error

**Figure 17:**
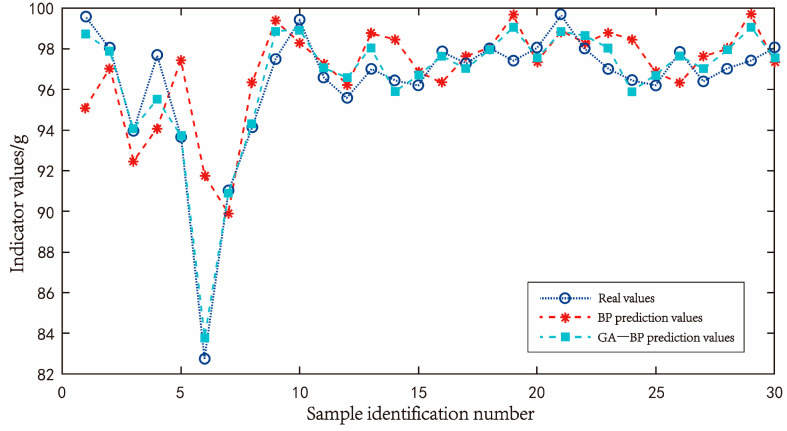
BP neural network predicted value, GA-BP neural network predicted value and true value relationship curve.

**Figure 18:**
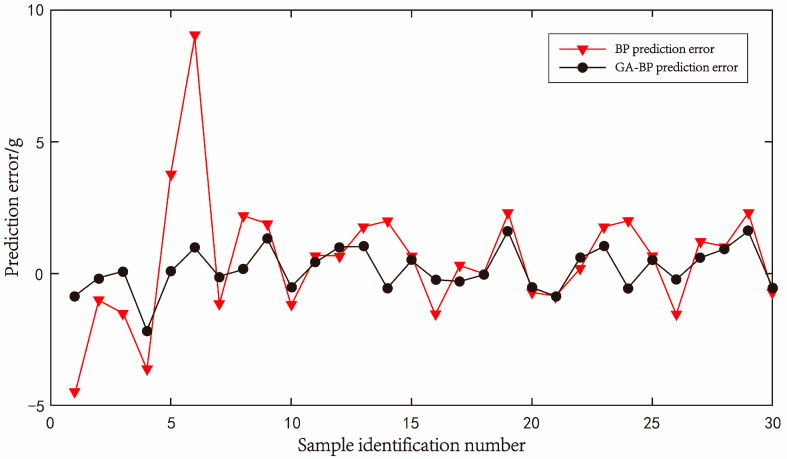
Error curve of prediction by BP neural network and GA-BP neural network.

Through the overall model training of the screened data, a total of 470 training groups and 30 test groups were set. The optimal number of hidden layer neurons was determined by trial and error. The optimal number of hidden layer neurons was determined to be 12 when the mean square error was the lowest. After the training of the neural network model, it can be seen that the goodness of fit of the two models is comparable through 30 groups of test data. The line diagram of the comparison between the predicted value and the real value is shown in Figure 17, and the line diagram of the prediction error of the two neural networks is shown in Figure 18.

Table 5 summarizes 30 sets of accuracy test data of the prediction model. The relative error values of the load cell output obtained through the neural network model are all less than 2%. The average absolute error values of the BP neural network and GA-BP neural network are 1.7571 and 0.67547, respectively, and the mean square error values are 5.94853 and 0.71361, respectively. Both kinds of neural network models can accurately predict the deviation of load cells according to the jitter condition.

After conducting a comparative analysis of the data collected, it can be concluded that the neural network model’s error elimination for the load cell sensor is highly effective, resulting in a significant reduction in errors. It can be seen more intuitively in Figure 18 that the accuracy predicted by the GA-BP neural network is higher. The jitter compensation scheme of the GA-BP neural network is finally adopted in this work.

#### 4.2.5. Analysis of the Final Results of the Calibration Compensation Experiment

**Figure 19:**
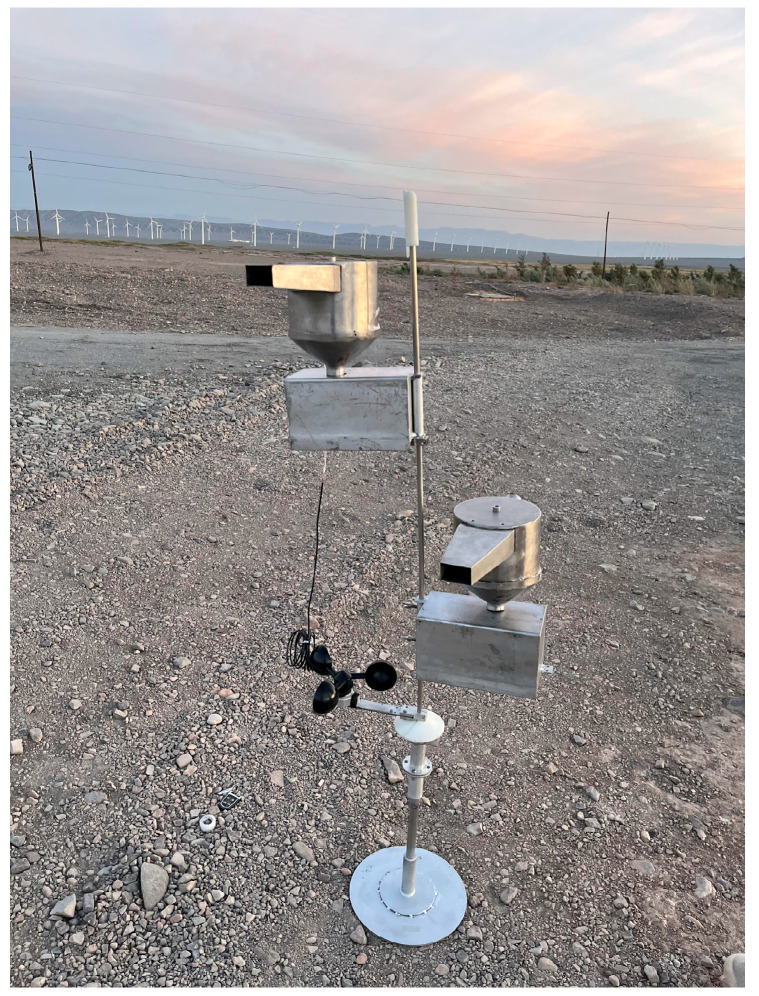
Field operation photo of the intelligent wind erosion monitoring system.

**Figure 20:**
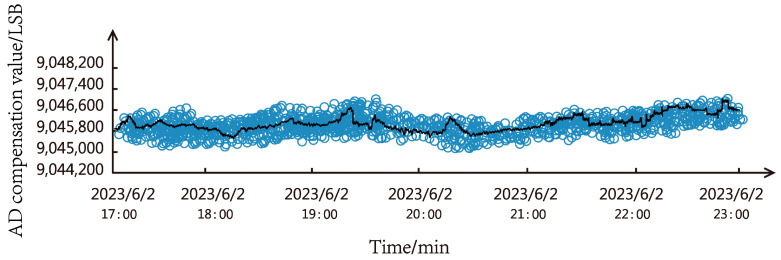
Line chart of the relationship between AD output value and measurement time.

We conducted a complete system operation test experiment for the entire system in the thirty-mile wind zone of Xiaocaohu, Turpan City, and the outdoor monitoring system was set up as shown in Figure 19. Under the circumstance of large temperature fluctuations and system jitter caused by strong winds, Figure 20 shows the line diagram of the relationship between the AD output value and the measurement time under 100 g standard weight. The blue small circles represent the output results of individual measurements during this time period, and the black line represents the average value of the measurement results within one minute. It can be seen from the diagram that the output value of the load cell is relatively stable, and the measured value of the load cell can be accurately output.

We comprehensively determined the specific values of the stability and accuracy improvement of the weighing sensor by analyzing the results of three experiments: the digital filtering experiment of the load cell, the temperature compensation experiment, and the jitter error elimination experiment. Stability is usually measured using the standard deviation, where a larger standard deviation indicates more dispersed data, and a smaller standard deviation indicates more concentrated data. This indicator is widely used in statistics to describe the stability and reliability of data.

In the digital filtering experiment, we calculated the standard deviation of 300 AD sampling points before and after median filtering. In the temperature error compensation experiment, we calculated the standard deviation of the average values obtained for seven different loads (no load, 5 g, 10 g, 20 g, 50 g, 100 g, 200 g) at intervals of 10 degrees from −20 degrees to 60 degrees. In the final jitter error elimination experiment, we first calculated the standard deviation for thirty test samples and then calculated the standard deviation for the results after the test samples were processed by the GA-BP neural network error elimination. The comparison of standard deviations before and after these three experiments determined an improvement in stability of 9.2%, 52.8%, and 35.2%, respectively. Taking the average of these three standard deviations, the overall output stability of the system was determined to have improved by 32.4%.

The utilized formula is depicted in Equation (1), where “Mean” represents the average of the dataset, and “xi” refers to the data point at position i within the dataset, and “n” is the total number of data points. This formula expresses the process of calculating the difference, square, and sum of squares of each data point with the mean value, ultimately taking the square root of the average to obtain the standard deviation.

(1) $$Standard\;deviation=\sqrt{\frac{\sum_{i=1}^{n}{\left(x_{i}-Mean\right)}^{2}}{n}}$$

The determination of system accuracy also involves analyzing the data from the three sets of experiments mentioned above. Through these experiments, the improvement in accuracy is calculated by taking the absolute difference between each data value and the expected value, then averaging the results. The individual improvements in accuracy for the three experiments are 10.2%, 55.8%, and 49.2%, respectively. After averaging, it is confirmed that the overall output accuracy of the system has improved by 38.4%. The percentages of improvement in stability and accuracy before and after the experiments are calculated using Equation (2).

(2) $$Percentage\;improvement=\frac{Value\;after\;the\;experiment-Value\;before\;the\;experiment}{Value\;before\;the\;experiment}\times 100$$

## 5. Discussion

Unlike traditional sand collecting instruments, this study mainly designs a fully automatic and high-precision soil erosion and desertification monitoring system. Researchers like Sherman and Goossens in the past mostly optimized the mechanical structure part of the sand collecting instrument to improve the efficiency of sand collection [17,18]. In order to obtain the wind erosion data more conveniently, and to reflect the whole process of wind erosion more objectively and accurately, providing data support for soil erosion research, researchers like Giménez, Antonio and Cui Yazhen have started to automate the sand collecting instrument in recent years [20,21]. However, the degree of automation has not met the needs of long-term outdoor operation, and there are no field test results. This study starts by improving the automation of the monitoring systems, and develops a soil erosion and desertification automatic monitoring system that can operate stably outdoors for a long time without manual operation. After the system was developed, it was found during the experiment that the core component, the load cell, will produce significant measurement errors under some specific environmental conditions. This problem seems to be an undeveloped research area. Although there are many studies on how to calibrate the load cell in the existing literature, this was the first time the system was placed in the sand trap equipment to work outdoors, especially to study the elimination method of errors caused by jitter in the load cell. No one has studied this method. This article, after referring to a large amount of the previous literature, conducts experiments on several factors that may cause errors in the equipment and designs error elimination plans for high-precision improvement. The experiment achieved good results and the overall accuracy has been greatly improved. In the process of designing and implementing, many areas that need improvement and further research have also been discovered. The soil erosion and desertification automatic monitoring system has completed several experimental tests, but in the design of the wind pressure error elimination scheme, due to limited experimental conditions, the wind blown into the sand collection unit is simulated as natural wind instead of wind with sand and dust. Experiments and follow-ups can use professional wind tunnel equipment to simulate the real outdoor wind and sand effects. The mechanical structure of the sand collection device was not sturdy during the test run, and the fixing device in the mechanical structure needs to be improved to minimize the external force interference and minimize the vibration of the equipment, to obtain better system accuracy. This is the focus of future research work, and we strive to put the designed monitoring system into practical use as soon as possible.

## 6. Conclusions

This work develops an intelligent wind erosion monitoring system, and completes the overall design of mechanical, design, and programming of the hardware circuit, realizing the fully automated operation of the equipment. Furthermore, the study has focused on the following aspects:

(1) Designed a collection speed reduction device to slow down the high-speed natural sand. Based on Ansys fluent, the collected deceleration part was simulated and the pressure generated by residual windblown sand on the load cell after deceleration was predicted. The accuracy of the estimated pressure value was analyzed in the simulation scenario, and the error caused by the wind pressure was eliminated, which improved the acquisition accuracy of the load cell.
(2) Developed a specific implementation plan for precision calibration of the load cell itself, and completed the sensor calibration experiment, temperature compensation experiment, simulation of natural wind jitter error elimination experiment, and the flexible use of median filter and cubic spline interpolation function. Real-time monitoring of the system’s jitter parameters was performed using the JY61 six-axis acceleration sensor. Two neural network models were trained in MATLAB 2021a software using jitter parameters, load cell measurement results, and load cell expected results. The best solution for eliminating jitter errors was determined by comparing the final error elimination effects. The accuracy calibration of the load cell was completed [32].

Finally, the field experiment was completed to measure the overall accuracy of the load cell, which verified that the accuracy of the device reached the expected standard and that the operation effect of the equipment was good. The development of this system improves the accuracy of wind erosion data acquisition and meets the requirements of high accuracy of wind erosion and desertification monitoring systems.

## Figures and Tables

**Table 1:** Factors and levels of orthogonal experiment.

Levels	Factors				
	(A) The Size of the Air Intake (mm × mm)	(B) The Diameter of the Cylindrical Diverter (mm)	(C) The Distance between the Top Point of the Tail Outlet and the Highest Point of the Equipment (mm)	(D) The Diameter of the Tail Outlet (mm)	(E) The Diameter of the Sand Outlet (mm)
1	45 × 25	40	19	18	30
2	45 × 30	60	39	23	40
3	50 × 25	80	59	28	50
4	50 × 30	100	79	33	60

**Table 2:** Orthogonal experiment results.

Test Number	A	B	C	D	E	Average Wind Speed at the Sand Outlet (m·s^−1^)
1	1	1	1	1	1	11.48
2	1	2	2	2	2	6.69
3	1	3	3	3	3	4.66
4	1	4	4	4	4	3.45
5	2	1	2	3	4	4.01
6	2	2	1	4	3	4.65
7	2	3	4	1	2	8.89
8	2	4	3	2	1	12.36
9	3	1	3	4	2	6.33
10	3	2	4	3	1	9.89
11	3	3	1	2	4	3.82
12	3	4	2	1	3	5.78
13	4	1	4	2	3	6.61
14	4	2	3	1	4	4.96
15	4	3	2	4	1	9.75
16	4	4	1	3	2	7.93

**Table 3:** Analysis of experimental results.

Factors	A	B	C	D	E
K1	26.28	28.43	27.88	31.11	43.48
K2	29.91	26.19	26.23	29.48	29.84
K3	25.82	27.12	28.31	26.49	21.7
K4	29.25	29.52	28.84	24.18	16.24
k1	6.57	7.11	6.97	7.78	10.87
k2	7.48	6.55	6.56	7.37	7.46
k3	6.46	6.78	7.08	6.62	5.43
k4	7.31	7.38	7.21	6.05	4.06
R	1.16	0.83	0.65	1.73	6.81

**Table 4:** Wind speed and pressure comparison table.

Average Wind Speed at the Air Intake (m·s^−1^)	Average Wind Speed at the Sand Outlet (m·s^−1^)	Average Wind Speed at the Tail Outlet (m·s^−1^)	Relative Pressure at the Sand Outlet (pa)
20	7.235	7.764	32.916
19	6.8	7.587	30.15
18	6.399	7.522	27.271
17	6.105	6.867	23.855
16	5.776	6.093	19.568
15	5.545	4.924	17.072
14	5.207	5.104	14.047
13	4.742	4.915	12.185
12	4.393	4.466	10.792
11	4.034	4.048	8.818
10	3.589	3.702	7.263
9	3.269	3.349	5.883
8	3.014	2.867	4.683
7	2.614	2.284	3.644
6	2.163	1.983	2.728
5	1.822	1.829	1.929
4	1.147	1.385	1.295
3	1.095	1.01	0.744
2	0.724	0.679	0.334
1	0.357	0.351	0.084

**Table 5:** BP neural network, GA-BP neural network predicted value, and error summary table.

Test Number	Experimental Values	BP Prediction Values	GA—BP Prediction Values	BP Prediction Error	GA-BP Prediction Error
1	99.561	95.087	98.716	−4.474	−0.845
2	98.042	97.038	97.866	−1.004	−0.176
3	93.967	92.457	94.050	−1.51	0.083
4	97.675	94.059	95.514	−3.616	−2.161
5	93.644	97.416	93.752	3.772	0.108
6	82.761	91.755	83.744	8.995	0.983
7	91.026	89.886	90.897	−1.139	−0.129
8	94.143	96.334	94.322	2.192	0.179
9	97.487	99.37	98.831	1.883	1.344
10	99.421	98.262	98.92	−1.159	−0.501
11	96.596	97.276	97.043	0.681	0.448
12	95.58	96.23	96.58	0.65	0.999
13	96.994	98.77	98.04	1.776	1.046
14	96.44	98.445	95.896	2.005	−0.544
15	96.189	96.869	96.706	0.68	0.517
16	97.845	96.314	97.624	−1.531	−0.221
17	97.292	97.618	97.006	0.326	−0.287
18	98.006	98.019	97.962	0.013	−0.044
19	97.407	99.713	99.031	2.306	1.624
20	98.054	97.358	97.535	−0.697	−0.519
21	99.689	98.822	98.832	−0.867	−0.857
22	98.014	98.225	98.627	0.21	0.612
23	96.994	98.77	98.04	1.776	1.046
24	96.44	98.445	95.896	2.005	−0.544
25	96.189	96.869	96.706	0.68	0.517
26	97.845	96.314	97.624	−1.531	−0.221
27	96.392	97.618	97.006	1.226	0.613
28	97.006	98.019	97.962	1.013	0.956
29	97.407	99.713	99.031	2.306	1.624
30	98.054	97.358	97.535	−0.697	−0.519

## Data Availability

Data are contained within the article.
